# Supplementary material for: MetaRibo-Seq measures translation in microbiomes
Source: Nat Commun. 2020 Jun 29;11:3268. doi: 10.1038/s41467-020-17081-z (PMC7324362; doi:10.1038/s41467-020-17081-z)
Supplement: Supplementary file 10 — Supplementary Data 7 [file 41467_2020_17081_MOESM10_ESM.zip › File2/Confidence_VeryHigh_Taxonomy/357860_out.krona.html]

Javascript must be enabled to view this page.

members
magnitude
magnitudeUnassigned
count
unassigned
taxon
rank

357860\_out

9

2
9
superkingdom

phylum
9
976

9
200643
class

order
171549
9

1
815
family

genus
816
1

species

SRS015794\_contig\_number\_5144
818
1

family
8
171552

838
8
genus

2
2293125

SRS019910\_contig\_number\_contig-100\_2949.128436SRS144537\_contig\_number\_40456
species

1
2292365
species

SRS049959\_contig\_number\_19895

species

SRS012849\_contig\_number\_8485
165179
1

1262925
2
species

SRS013521\_contig\_number\_8469SRS013521\_contig\_number\_9141

species

SRS015794\_contig\_number\_5520SRS078176\_contig\_number\_1158
159272
2
